# Assessment of Vitamin D Status and Calcium–Phosphate Metabolism in Patients with Hypophosphatasia—A Single-Center Cohort Study

**DOI:** 10.3390/nu18193119

**Published:** 2026-09-22

**Authors:** Izabela Michałus, Marta Pejska, Aleksandra Jóźwiak-Markiewicz, Agnieszka Konopka, Ewa Starostecka, Aneta Gwozdowska, Arkadiusz Zygmunt

**Affiliations:** 1 Department of Endocrinology and Metabolic Diseases, Polish Mother’s Memorial Hospital—Research Institute, 93-338 Lodz, Poland; aleksandra.jozwiak-markiewicz@iczmp.edu.pl (A.J.-M.); agnieszka.konopka@iczmp.edu.pl (A.K.); ewa.starostecka@iczmp.edu.pl (E.S.); arkadiusz.zygmunt@iczmp.edu.pl (A.Z.); 2 Outpatient Clinic for Congenital Metabolic Diseases in Children, Polish Mother’s Memorial Hospital—Research Institute, 93-338 Lodz, Poland; aneta.gwozdowska@iczmp.edu.pl; 3 Department of Developmental Age and Adult Endocrinology, Medical University of Lodz, 93-338 Lodz, Poland

**Keywords:** hypophosphatasia, rare disease, vitamin D, calcium–phosphate metabolism

## Abstract

**Background:** Hypophosphatasia (HPP) is a rare genetic disorder caused by a loss-of-function mutation in the *ALPL* gene, which encodes tissue-nonspecific alkaline phosphatase (TNSALP). Reduced TNSALP activity in HPP primarily leads to bone mineralization disorders. Given the complex and challenging nature of issues related to vitamin D and HPP, it is essential to evaluate each patient individually and adjust the appropriate dose of vitamin D according to their specific needs in order to maintain calcium phosphate homeostasis and normal vitamin D levels. Maintaining proper vitamin D homeostasis in patients with hypophosphatasia is one of the greatest challenges. **Objective:** The objective of this study was to conduct a retrospective, comprehensive assessment of calcium–phosphate metabolism, with particular emphasis on vitamin D status in patients with genetically confirmed hypophosphatasia (HPP), and to identify potential correlations between 25(OH)D concentrations and other parameters of calcium–phosphate in this group of patients. **Methods:** A single-center, retrospective pilot study was conducted in a small cohort (n = 36) of patients suffering from an ultra-rare genetic disorder, hypophosphatasia. The small sample size reflects the ultra-rare nature of the disease; so far, HPP has been diagnosed in approximately 50 patients in Poland (prevalence 1:300,000). The analysis included medical records of patients. **Results:** Vitamin D deficiency was not observed in the majority of patients with hypophosphatasia in the study group. The initiation of long-term enzyme replacement therapy (with asfotase alfa) did not significantly affect the annual change in mean serum vitamin D concentration. As established in the literature, growth disorders are an inherent feature of the clinical picture of hypophosphatasia. This was reflected in our study group, where nearly half of the pediatric patients achieved a height below the 10th percentile. **Conclusions:** Within this limited sample, these growth issues did not appear to be associated with disturbances in calcium and phosphate metabolism or vitamin D deficiency.

## 1. Introduction

Hypophosphatasia (HPP) is a rare genetic disorder caused by a loss-of-function mutation in the *ALPL* gene, which encodes tissue-nonspecific alkaline phosphatase (TNSALP). This congenital metabolic bone disorder is inherited in an autosomal recessive or dominant manner [1]. The pathogenic variants underlying HPP are located on the short arm of chromosome 1 (1p36.1-34) [2,3]. Reduced TNSALP activity in HPP primarily leads to bone mineralization disorders, including the pathological accumulation of inorganic pyrophosphate (PPi), a potent inhibitor of mineralization. Physiologically, TNSALP is responsible for the extracellular dephosphorylation of inorganic pyrophosphate (PPi) to the inorganic form of phosphate (Pi). The proper course of this reaction is essential for the correct formation of hydroxyapatite in the bone matrix, and this entire process occurs in the cell membranes of hypertrophic chondrocytes and osteoblasts [4].

The epidemiology of HPP is characterized by significant geographical and population-based variation. This characteristic stems from the large number of known mutations in the *ALPL* gene (over 400). It is estimated that severe, life-threatening perinatal and infantile forms occur with a frequency of up to approximately 1 in 300,000 live births in European populations [5]. In contrast, milder forms detected at a later age (childhood, adult, and odontohypophosphatasia) are much more common. Studies involving molecular diagnostics show that the prevalence of carriers of pathogenic variants of the mild HPP phenotype may reach as high as 1:6370. Atypical/milder forms may still go undetected, and the prevalence of heterozygous pathogenic *ALPL* variants is estimated at 1:80 live births [6]. The epidemiological statistics cited demonstrate that a significant proportion of patients with the adult or childhood form remain undiagnosed or are treated for years due to misidentified metabolic osteopathies of a different etiology [5,6,7].

Despite growing knowledge among physicians of various specialties and an increasingly better understanding of rare diseases, establishing an accurate diagnosis of HPP often involves a diagnostic process spanning many years [8]. In children, the bone changes characteristic of HPP are often mistaken for congenital bone fragility, classic deficiency rickets, vitamin D-resistant rickets, or hypophosphatemic rickets, whereas in the adult population, recurrent fractures are sometimes interpreted as early osteoporosis or osteomalacia [9]. In patients suffering from chronic musculoskeletal pain, the condition is often misdiagnosed as fibromyalgia [10]. Delayed diagnosis, and consequently delayed targeted enzymatic therapy for HPP, leads to the development of systemic complications, not limited to the musculoskeletal system [11]. The accumulation of pyrophosphates directly contributes to the disruption of the architecture of epiphyseal cartilage, which may contribute to short stature in patients [12]. The absence of normal apoptosis of hypertrophic chondrocytes in the growth plates leads to widening and remodeling of the epiphyses, which is clinically similar to rickets [13,14].

Skeletal mineralization disorders and biochemical abnormalities observed in the course of HPP necessitate monitoring of the factors regulating calcium–phosphate homeostasis, among which vitamin D plays a particularly important role.

Vitamin D is the only vitamin endogenously synthesized by human skin; however, cutaneous synthesis is often insufficient to meet the body’s needs, especially during the fall–winter season, with limited exposure to UVB radiation, or in latitudes with low sunlight. Vitamin D is one of four fat-soluble vitamins; however, obtaining an adequate amount of vitamin D3/D2 from the diet is extremely difficult, which is why most patients, including those with HPP, require year-round supplementation with a prophylactic dose in accordance with recommendations [15]. Vitamin D performs many essential functions in the body; acting through the VDR receptor, it is one of the key factors regulating bone turnover. This is particularly important in patients with HPP, who are known to have a tendency toward hypercalcemia and hypercalciuria [16].

Given the complex and challenging nature of issues related to vitamin D and HPP, it is essential to evaluate each patient individually and adjust the appropriate dose of vitamin D according to their specific needs in order to maintain calcium–phosphate homeostasis and normal vitamin D levels [15]. Failure to monitor vitamin D levels through laboratory testing and uncontrolled supplementation with cholecalciferol and/or calcium may increase the risk of nephrocalcinosis [16].

Maintaining proper vitamin D homeostasis in patients with hypophosphatasia is one of the greatest challenges in modern osteology, as it presents a kind of clinical paradox. The incorporation of calcium and phosphorus into bone is impaired due to a congenital enzyme deficiency, which is why these patients often exhibit a predisposition to hypercalcemia [16]. An abnormal intake of vitamin D, along with supplementation, may contribute to increased renal tubular calcium reabsorption [16]. The resulting hypercalciuria and significant precipitation of calcium salts in the renal tubules may lead to the development of nephrocalcinosis [16]. However, it should be noted that chronic vitamin D deficiency also has negative consequences [15]. A 25(OH)D deficiency deprives the skeletal system of a fundamental regulatory substrate that stimulates the parathyroid glands to increase secretion of parathyroid hormone (PTH) [17].

The development of secondary hyperparathyroidism exacerbates osteoclastogenesis and bone resorption. In a patient whose skeleton is already undergoing primary demineralization due to TNSALP deficiency, such secondary disorders further contribute to the progression of skeletal complications [15], which compels clinicians to seek an appropriate vitamin D concentration that is high enough to suppress PTH secretion, yet still does not exacerbate hypercalciuria [17]. For HPP, there is still a lack of clear evidence regarding the 25(OH)D levels that should be maintained and the recommended supplementation doses to avoid exacerbating the aforementioned processes [15].

The wide range of clinical presentations and mineral metabolism disorders in patients with HPP necessitates the monitoring of calcium–phosphate balance and imaging results [18,19]. The basic panel includes measurements of inorganic phosphate, calcium, PTH, ALP, and vitamin D in the blood, as well as monitoring of the Ca/Cr ratio, which is calculated based on calcium and creatinine concentrations in the urine sample. In addition to determining the potential presence of hypercalcemia and hypocalcemia, it is also important to evaluate kidney function via ultrasound and assess bone mineral density using the DEXA method. The introduction of targeted enzymatic replacement therapy (asfotase alfa) has significantly improved patient prognosis [20,21], but the impact of causal therapy on long-term vitamin D requirements remains an area for further exploration.

**Main Objective**

The study aim was to conduct a retrospective, comprehensive assessment of vitamin D status, including calcium and phosphorus metabolism, in patients with genetically confirmed hypophosphatasia (HPP), as well as to identify potential correlations between 25(OH)D levels and other biochemical and anthropometric parameters.

**Specific objectives**

1. Determining baseline cholecalciferol status profile in the entire study group of patients with HPP.
2. Analysis of changes in serum 25(OH)D levels and selected biochemical parameters and markers of bone turnover (in particular, alkaline phosphatase activity and PTH levels) in patients treated with enzymatic substitution therapy with asfotase alfa.
3. Identification and assessment of the strength of correlation between vitamin D concentration and key biochemical indicators in blood and urine (total calcium, inorganic phosphate, magnesium, alkaline phosphatase, PTH, and the Ca/Cr ratio) and anthropometric parameters in the pediatric population with HPP.
4. Assessment of somatic development (based on height/length centile charts and body mass index—BMI) in the pediatric subpopulation of the study group, combined with the verification of the co-occurrence of growth disorders with possible vitamin D deficiencies and identification of the prevalence of short stature in this group.

## 2. Materials and Methods

### 2.1. Study Design

A single-center, retrospective study was conducted in patients suffering from a rare genetic disorder—hypophosphatasia. The analysis included medical records of patients under the care of the Department of Endocrinology and Metabolic Diseases and the Clinic for Inborn Errors of Metabolism at the Polish Mother’s Memorial Hospital—Research Institute (PPMH-RI) in Lodz, Poland, with a diagnosis of HPP established on the basis of genetic testing (confirmed mutation in the *ALPL* gene). The analysis was performed using anonymized data collected from routine clinical practice related to patient care and management. The study was approved by the Bioethics Committee of the PPMH-RI; approval number KB-32/2026.

### 2.2. Study Population

The inclusion criteria for patients in the study were the presence of a mutation in the *ALPL* gene and access to medical records at specified time points for selected patients. From the study cohort (n = 36), four subgroups were identified based on age and the initiation of enzyme replacement therapy (ERT). Two study groups were identified based on age: the pediatric population (<18 years of age; n = 21) and adults (≥18 years of age; n = 15). The other two groups were defined based on the implementation of enzyme replacement therapy (ERT): asfotase alfa (n = 13; including adults n = 3). Patients received ERT at a dose of 2 mg/kg body weight three times a week. Data analysis for patients in the treatment group was conducted at two predefined time points: baseline (immediately prior to the initiation of ERT) and follow-up (after 12 months of continuous therapy); due to the longer duration of therapy extending beyond the defined baseline, three patients (designated in the study as HPP_01, HPP_02, and HPP_03) were excluded from the statistical analysis conducted in this group (baseline, n = 13 vs. follow-up, n = 16) and were only included in the cross-sectional analysis of parameters at follow-up (n = 16). The cohort also included a control group of patients not treated with ERT who had also been under the center’s care in recent years (n = 20).

### 2.3. Study Data and Clinical and Auxological Assessment of the Study Group

Study data were obtained from electronic medical records and physical hospital records. The data used for analysis included demographic parameters such as age and sex, as well as data from blood and urine laboratory tests. The auxological assessment only included the pediatric cohort (insufficient data in the adult patient group to perform statistical analysis) and was based on the assessment of body weight, height/body length, and the calculated BMI (kg/m^2^). The obtained anthropometric parameters were referenced to centile charts based on the results of the OLA and OLAF studies for the Polish population, standardizing the values of body weight, height/body length, and BMI. Short stature was defined in this group as a height below the 3rd centile for a given age and sex.

### 2.4. Biochemical Parameters

Biological samples (blood serum, first-morning urine) were collected on an empty stomach under standard conditions; no calcium loading was administered. The biochemical panel used in this study included measurements of 25-hydroxyvitamin D [25(OH)D], total calcium (Ca), inorganic phosphorus (P), magnesium (Mg), parathyroid hormone (PTH), and total alkaline phosphatase (ALP).

To determine the risk of hypercalciuria in patients, the calcium-to-creatinine ratio (Ca/Cr; mg/dL/mg/dL) was calculated based on measured concentrations of calcium (Ca-U) and creatinine (Cr-U) in the morning urine sample.

Vitamin D status, based on serum 25(OH)D concentrations, was categorized according to the following criteria:

- Deficiency < 20 ng/mL;
- Suboptimal concentration 20–30 ng/mL;
- Optimal concentration 30–50 ng/mL;
- High concentration 50–100 ng/mL;
- Potentially toxic concentration >100 ng/mL.

### 2.5. Statistical Analysis

Statistical analysis of the collected clinical data was performed using R/RStudio (R version 4.6.0 executed via RStudio-version 2026.05.0). The normality of the distribution of continuous variables was verified using the Shapiro–Wilk test. Due to the small sample sizes of the analyzed groups and the failure to meet the assumptions of parametric tests, continuous variables were presented primarily as the median with the interquartile range (IQR), and for descriptive purposes additionally as the arithmetic mean with the standard deviation (±SD). Categorical variables were presented as frequencies and percentages.

The main longitudinal analysis involved a comparison of 25(OH)D, ALP, and PTH concentrations between time points baseline and follow-up in 12 patients with complete paired data. The Wilcoxon signed-rank test was used to compare results at the two time points.

The Mann–Whitney test was used for comparisons between two independent groups, including children vs. adults, women vs. men, and treated vs. untreated patients. Intergroup analyses were performed separately for the time points at baseline and follow-up, provided that both groups being compared were available at a given time point.

Associations between 25(OH)D concentration and selected clinical and laboratory parameters were assessed using Spearman’s rank correlation coefficient. The main correlation analyses included relationships between 25(OH)D and ALP and PTH at baseline and follow-up, as well as relationships between changes in 25(OH)D concentration and changes in ALP and PTH over time.

In all analyses, a *p*-value of <0.05 was set as the threshold for statistical significance. Given the exploratory nature of the study and the small sample size, the results were interpreted with caution, taking into account the size of the subgroups analyzed as well as the direction and strength of the observed associations.

## 3. Results

### 3.1. Demographic and Clinical Characteristics of the Cohort

The study included 36 patients diagnosed with hypophosphatasia (HPP), including 21 children and 15 adults. The mean age of the patients was 21.8 years. In the analyzed group, females slightly outnumbered males (female n = 20; male n = 16). In terms of the clinical phenotype of HPP, the childhood form of hypophosphatasia predominated (n = 23). The adult form (n = 8) and odontohypophosphatasia (n = 3) were observed much less frequently in the study participants, while the most severe forms involved single cases (perinatal form n = 1; infantile form n = 1). During the analyzed period, 16 patients underwent targeted treatment.

### 3.2. Vitamin D Status

The mean baseline 25(OH)D concentration calculated for the entire study group was 34.2 ng/mL. According to the accepted classification of vitamin D levels, the vast majority of patients with available data at the specified time point (n = 33) had optimal (n = 16) or suboptimal (n = 12) concentrations. Deficiency was found in only two patients; in contrast, elevated levels were observed in only three out of 33 patients. After treatment (follow-up; n = 15), the mean concentration was 39.2 ng/mL; optimal (n = 9) and suboptimal (n = 3) concentrations were observed in the majority of patients, and no toxic levels were observed in any patient (Table 1).

In order to accurately assess the effect of treatment on vitamin D levels, a subgroup of patients with complete pairs of results from both time points was identified from the entire cohort. The assessment of changes in vitamin D concentrations in the group of patients treated with ERT was conducted using a longitudinal analysis aimed at comparing serum 25(OH)D concentrations at baseline and after 12 months of therapy (follow-up).

The final statistical analysis was performed in a subgroup of 12 patients (HPP_04-HPP_15) with complete pairs of matched vitamin D concentration results.

The mean 25(OH)D concentration in the study group was initially 41.98 (±24.55) ng/mL, while after 12 months it decreased by a statistically insignificant margin, reaching the value of 39.75 (±16.16) ng/mL.

Although an individual analysis of the trajectory of changes indicates an upward trend in some of the subjects, due to high individual variability, the results indicate that long-term treatment with asfotase alfa does not disrupt vitamin D homeostasis. While fluctuations in 25(OH)D levels were observed during therapy, these variations generally remained within or shifted toward optimal clinical target ranges. For instance, the most pronounced decrease was seen in patient HPP_13, which actually represented a positive normalization from a highly elevated baseline (114.9 ng/mL) to an optimal level (45.9 ng/mL). It is important to emphasize that, throughout the treatment period, patients received routine prophylactic vitamin D supplementation, with daily doses individually adjusted according to their age and current clinical guidelines (1000 IU for children, 2000 IU for adults). Therefore, our findings suggest that concurrent enzyme replacement therapy does not interfere with the effectiveness of standard vitamin D supplementation and does not necessitate aggressive dosage modifications.

Table 2 presents the descriptive data of the overall vitamin D status in the entire study cohort (n = 36), including a breakdown by pediatric and adult patients. The complete profile of 25(OH)D concentrations for all patients is presented below, revealing very high individual variability. The complete profile of 25(OH)D concentrations for all patients, divided into baseline and follow-up after one year, is presented in Table 2. The summary of the data reveals very high individual variability in vitamin D concentrations among patients with HPP. Baseline results were characterized by a wide range—from values close to deficiency (approx. 20 ng/mL) to extremely high concentrations of 114.9 ng/mL.

The histogram of baseline 25(OH)D concentrations shows a clear right-skewed distribution, with the majority of recorded results falling below 50 ng/mL. The vertical dashed line marks the group median at 31.10 ng/mL, while the isolated bar indicates the presence of a single outlier in the study cohort (Figure 1).

A comparison of the two time points reveals a marked change in the distribution of 25(OH)D concentrations, where the strong initial right-skewed asymmetry and the presence of an outlier at baseline disappear after one year of observation. At follow-up, the distribution becomes more symmetrical and unimodal, with most measurements concentrated in the range between 25 and 40 ng/mL, which correlates with the attainment of normal distribution characteristics in the final stage of the analysis (Figure 2).

Due to the fact that the baseline distribution of vitamin D concentrations deviated significantly from a normal distribution (Shapiro–Wilk test, *p* < 0.001), which was due, among other things, to the presence of an outlier at 114.9 ng/mL, median analysis and nonparametric tests were used for further intergroup comparisons (Figure 3, Figure 4 and Figure 5).

At baseline, the distribution of 25(OH)D concentrations was similar in both subpopulations, although the median in the treated group was slightly higher than in the untreated patients. In the cohort receiving ERT, a single extreme outlier of 114.9 ng/mL was noted, whereas in untreated patients, individual measurements showed less variation (Figure 3).

The median 25(OH)D concentrations were similar in both sexes, with greater individual variability and the presence of a single outlier being observed in the male group. After 12 months (follow-up), the median values increased slightly in both subgroups, showing a very similar final distribution profile of vitamin D concentrations in women and men (Figure 4).

In the presented groups of children and adults, the median vitamin D concentrations were similar; however, greater variability was observed in the pediatric group. At follow-up, the median levels increased in both age groups, with the adult subpopulation characterized by minimal variability due to the limited number of endpoints, while a wide range of individual values persisted in children (Figure 5).

### 3.3. Evaluation of Biochemical Parameters

In the baseline analysis of calcium–phosphate metabolism markers (baseline), the mean serum concentrations were as follows: total calcium 2.49 mmol/L, inorganic phosphate 1.58 mmol/L, and magnesium 0.85 mmol/L. The mean calcium-to-creatinine ratio (Ca/Cr, calculated based on Ca-U and Cr-U) in urine was 0.156. At the time point (follow-up) following ERT administration, a highly statistically significant increase in mean alkaline phosphatase activity was observed (from 47.46 U/L to 8101.87 U/L; *p* < 0.001), which directly reflects the supply of exogenous enzyme (asfotase alfa) in the treated subpopulation.

As detailed in Table 3, changes in mean calcium, phosphorus, and magnesium concentrations between baseline and follow-up evaluations were not statistically significant (calcium: 2.49 vs. 2.55 mmol/L, *p* = 0.104; phosphorus: 1.58 vs. 1.74 mmol/L, *p* = 0.515; magnesium: 0.85 vs. 0.86 mmol/L, *p* = 0.753).

Descriptive statistics and assessment of the normality of distribution (Shapiro–Wilk test) for the main vitamin D levels in the study cohort. Q1—first quartile (25th percentile); Q3—third quartile (75th percentile); Δ—difference in values between follow-up and baseline; statistically significant results are highlighted in bold (*p* < 0.05). The Wilcoxon signed-rank test was used to assess the statistical significance of changes in the concentrations of the studied parameters between baseline and follow-up in the subgroup of patients with complete data pairs (n = 12). Data are presented as the median and interquartile range (Q1–Q3). No significant changes were observed for vitamin D concentration after one year of follow-up (median at time point baseline: 34.9 ng/mL [Q1–Q3: 31.03–39.90] vs. follow-up: 37.85 ng/mL [Q1–Q3: 30.85–4.93]; *p* = 0.969; median Δ: −2.05 ng/mL) (Table 4).

### 3.4. Somatic Development of Pediatric Patient

The somatic development and nutritional status of the pediatric patients (n = 18) were assessed based on height and body mass index (BMI) percentile charts.

A comparison of anthropometric parameters with 25(OH)D status did not reveal a coexistence of short stature with vitamin D deficiency in our study group; patients with growth retardation were characterized by optimal vitamin D status. It is worth noting that hypophosphatasia is an ultra-rare condition, and the analyzed cohort represents the vast majority of diagnosed cases in Poland. Consequently, these findings provide a unique and highly valuable clinical perspective on the natural course of the disease in this specific population.

The somatic development of the pediatric population was assessed, among other factors, based on the patients’ height/length relative to percentile charts (n = 18). The results are presented in Figure 6.

Half of the study group (n = 9; 50%) fell within the broad population norm, with height ranging between the 10th and 90th percentiles. However, the very high proportion of patients with marked growth disorders is noteworthy. As many as one-third of the patients (n = 6; 33.3%) exhibited short stature, defined as a height below the third percentile. Additionally, in another two patients (11.1%), height was bordering on short stature, falling within the narrow range between the third and 10th percentiles.

These results indicate that nearly half of the entire study cohort (n = 8; 44.4%) were below the 10th percentile in terms of development for their age and sex. Such a significant proportion of patients with growth deficiency clearly reflects the systemic impact of hypophosphatasia on growth and skeletal mineralization processes. In the study group, only one patient (5.6%) was noted to have a height in the 90th–97th percentile range, and none of the patients exceeded the 97th percentile.

The nutritional status of pediatric patients was assessed based on body mass index (BMI) relative to age- and sex-specific percentile charts (n = 18). The results of the analysis are presented in Figure 7. In contrast to previously observed growth disorders, not a single case of underweight was recorded in the study group (no cases below the 10th percentile were noted). In the majority of patients (n = 12; 66.7%), nutritional status was normal, and their BMI fell within the range between the 10th and 90th percentiles.

A BMI indicating overweight was observed in one in three patients in the presented group (n = 6; 33.3%). Four subjects (22.2%) had a BMI between the 90th and 97th percentiles (a result indicating overweight). In two patients from this group (11.1%), the BMI was >97th percentile, corresponding to obesity. These figures highlight the fact that despite the common occurrence of short stature among patients, their nutritional status remains normal.

Spearman’s rank correlation test (rs) was used to assess the correlation between vitamin D concentration (at baseline, follow-up, and for its change) and clinical, biochemical, and anthropometric parameters (Table 5). To minimize the risk of false-positive results from multiple testing, the Benjamini–Hochberg correction (FDR) was applied.

The results of the statistical analysis revealed three correlations that reached the threshold of statistical significance (*p* < 0.05). At baseline, a strong negative correlation was found between vitamin D concentration and the calcium-to-creatinine ratio (rs = −0.692; *p* = 0.009) and a weak positive correlation with serum inorganic phosphate (rs = 0.376; *p* = 0.031). Results close to the significance threshold were also observed at follow-up for the correlation between 25(OH)D and inorganic phosphate concentration (rs = −0.486; *p* = 0.066) and between 25(OH)D and height (rs = 0.524; *p* = 0.080). After applying the FDR correction, none of the observed associations remained statistically significant (adjusted *p* > 0.05).

Loss of statistical significance is a common consequence of applying rigorous statistical controls to a small sample size, which is an unavoidable limitation when analyzing patients with rare diseases.

The correlation between changes in the studied parameters (difference between follow-up and baseline) was also assessed. Changes in vitamin D concentration did not correlate with changes in bone turnover markers (ALP, PTH) or ion concentrations (Ca, P, Mg). These parameters underwent modifications during the one-year follow-up period independently of changes in 25(OH)D concentration.

## 4. Discussion

The natural course of hypophosphatasia as an ultra-rare disease is currently the subject of extensive research. Due to potential biochemical abnormalities, particularly in severe forms (perinatal and infantile), patients with hypophosphatasia require monitoring of calcium and phosphate levels and vitamin D concentrations [22]. In biochemical tests, abnormalities most commonly occur in severe forms of HPP (perinatal and infantile). In these forms, hypercalcemia, hyperphosphatemia, hypercalciuria and secondary hypoparathyroidism are observed. In the group of HPP patients analyzed in this study, no significant disturbances in calcium–phosphate metabolism were found, which was likely due to the fact that the majority were diagnosed with a milder type of HPP—the childhood or adult form. Two patients with severe forms (perinatal and infantile) had been under care and receiving treatment for many years; at the time of assessment, they showed no disturbances in calcium–phosphate metabolism [23,24,25,26,27].

For many years, knowledge of hypophosphatasia as an ultra-rare disease was limited, due to the fact that there were few patients with this diagnosis, and most publications consisted of isolated case reports. It was only following the introduction of the Global HPP Registry that the natural course of the disease began to be understood on the basis of the data collected. The studies published to date have primarily analyzed clinical symptoms, patients’ genotypes and their quality of life. There are few studies in the literature reporting on biochemical abnormalities, including vitamin D status [27].

It is generally accepted that routine measurement of serum 25(OH)D levels is not indicated in the general population. However, in metabolic disorders in which vitamin D plays an important role in calcium–phosphate homeostasis, its status should be closely monitored. In hypophosphatasia, adequate vitamin D levels should be maintained and deficiency prevented. Nevertheless, excessive vitamin D supplementation is not recommended, as it may exacerbate hypercalcemia and hypercalciuria, particularly in patients with more severe forms of HPP.

Among the patients with hypophosphatasia included in our study, all individuals receiving enzyme replacement therapy were administered prophylactic doses of vitamin D. Our analysis demonstrated that this approach allowed optimal 25(OH)D concentrations to be maintained in the majority of patients. Patients treated with enzyme replacement therapy within the drug program undergo regular monitoring of calcium–phosphate metabolism parameters, including the measurement of serum 25(OH)D levels. This enables the early detection of metabolic abnormalities and facilitates appropriate therapeutic adjustments when necessary.

Our findings are consistent with those reported by Whyte et al., who observed normal serum 25(OH)D concentrations in a cohort of 16 patients with various forms of HPP [28].

It is also worth noting that the analysis of anthropometric parameters, which shows that short stature (height < the third percentile for gender and age) was diagnosed in six patients, whilst in a further two patients it fell within the 3rd–10th percentile range. A parallel analysis of the BMI in this group revealed no cases of underweight; 12 children had a normal BMI, whilst the remaining six patients (33%) were diagnosed with obesity. At the same time, no association was observed between short stature and vitamin D deficiency—patients with growth retardation had optimal vitamin D levels, suggesting that the cause lies in genetic factors rather than vitamin D deficiency.

In a study by Hogler et al. based on data from the Global Hypophosphatasia Registry, it was shown that the height of children with HPP was significantly lower than the MPH target value (−0.66 standard deviations); however, short stature (<3rd percentile) was observed in only a small number of patients. Furthermore, children with perinatal/infantile HPP were shorter for their age compared with children in whom HPP developed during adolescence [29].

The finding that as many as 33% of the patients described in our study are obese provides a basis for further research. An interdisciplinary approach to patient care, including dietary management, may prove beneficial in improving care for patients with HPP. An important observation is that the dietary intake of calcium and vitamin D in patients with rare bone diseases is too low to meet the body’s requirements. In this group of patients, the co-occurrence of overweight and obesity should be an indication for nutritional assessment, dietary counseling and regular monitoring of body composition [30].

## 5. Conclusions

Based on a retrospective analysis of clinical, biochemical and auxological parameters in patients with hypophosphatasia (HPP), the following observations were made.

Vitamin D deficiency is not observed in the majority of patients with hypophosphatasia. Baseline 25(OH)D concentrations in the study cohort are characterized by high inter-individual variability; however, in the vast majority of cases, they remain at suboptimal or optimal levels, suggesting the satisfactory efficacy of standard supplementation, including cases where enzyme treatment is initiated.

The initiation of long-term enzyme replacement therapy (with asfotase alfa) does not significantly affect the annual change in mean serum vitamin D concentration. However, a reduction in the standard deviation is observed, indicating that the treatment may potentially contribute to stabilizing vitamin D homeostasis by preventing significant fluctuations.

Although asfotase alfa does not directly interact with the enzymes responsible for vitamin D metabolism, it indirectly influences this pathway by reestablishing physiological bone mineralization and normalizing overall mineral balance and homeostasis. Consequently, the introduction of targeted enzyme replacement therapy (ERT) in hypophosphatasia profoundly shifts the systemic utilization of calcium, phosphorus, and vitamin D. As expected, we observed that the initiation of ERT induces significant longitudinal changes in selected markers of bone metabolism (e.g., a decrease in PTH levels and an increase in ALP activity, reflecting the action of the exogenous enzyme). Importantly, our correlation analysis indicates that despite these profound metabolic shifts, the changes occur independently of fluctuations in 25(OH)D concentrations. This further supports the conclusion that standard prophylactic supplementation remains sufficient to maintain optimal vitamin D status during ERT, even as the body undergoes rapid skeletal remineralization [31].

As established in the literature, growth disorders are an inherent feature of the clinical picture of hypophosphatasia, which was reflected in our study group, with nearly half of the pediatric patients achieving a height below the 10th percentile. In this comprehensive national cohort, these growth issues occurred independently of disturbances in calcium and phosphate metabolism or vitamin D deficiency. Although statistical generalizations are inherently limited by the ultra-rare nature of the disease, these findings provide robust real-world evidence supporting the pathophysiological, rather than nutritional, origin of short stature in patients with HPP. This finding may provide a focus for further research aimed at determining the metabolic phenotype of patients with hypophosphatasia, analyzing body composition, and examining dietary patterns.

## Figures and Tables

**Figure 1:**
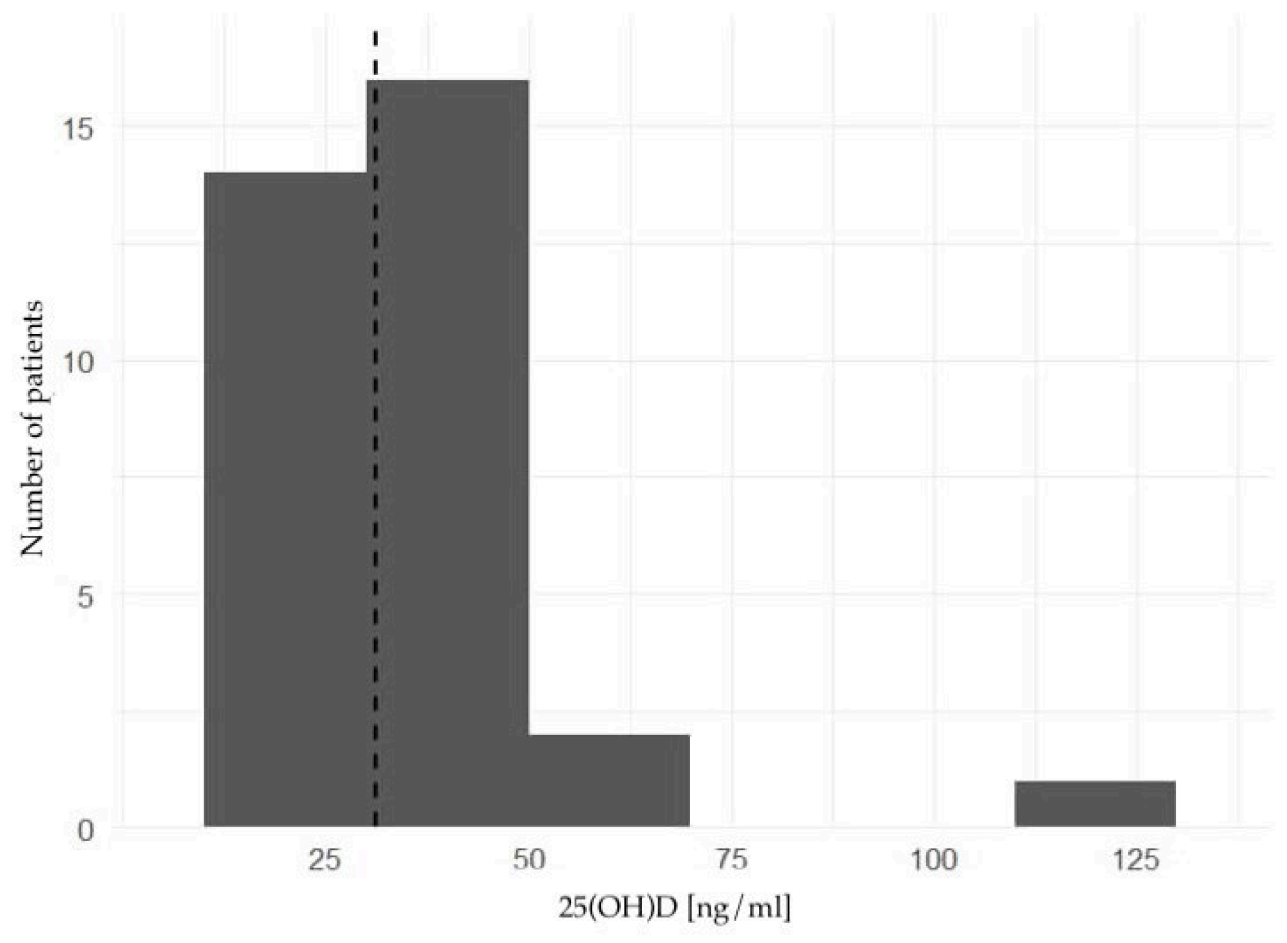
Vitamin D levels in all patients at baseline.

**Figure 2:**
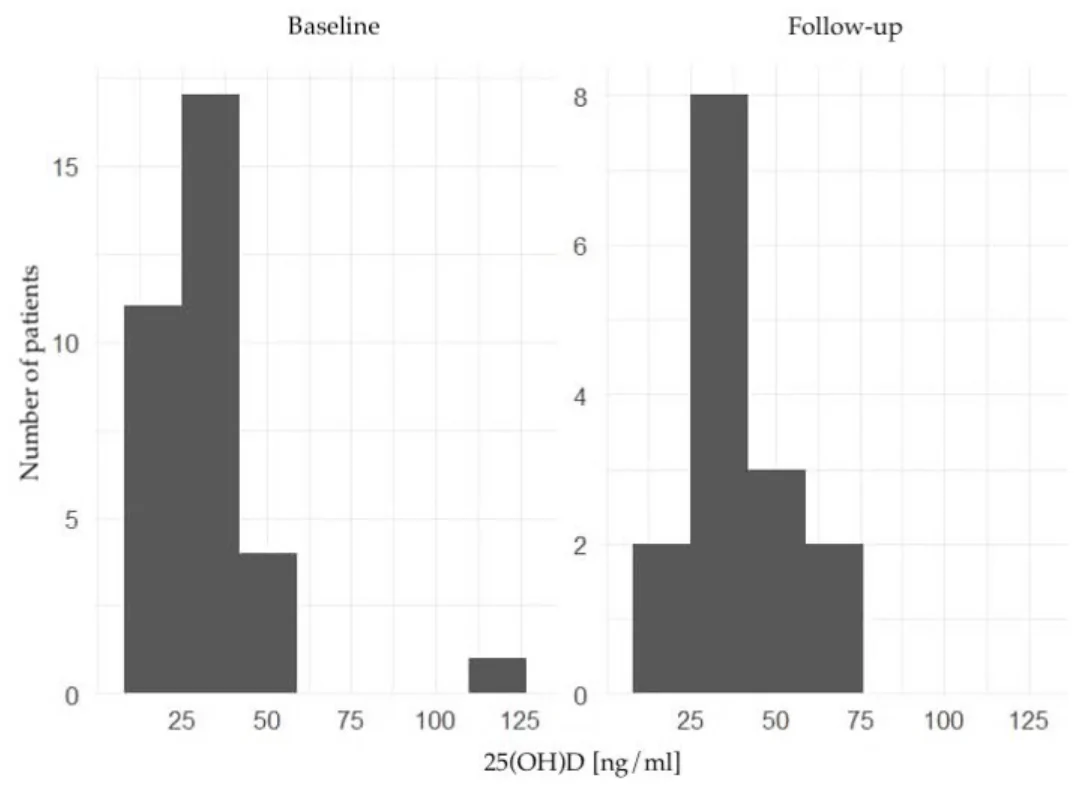
Vitamin D levels in all patients at baseline and follow-up.

**Figure 3:**
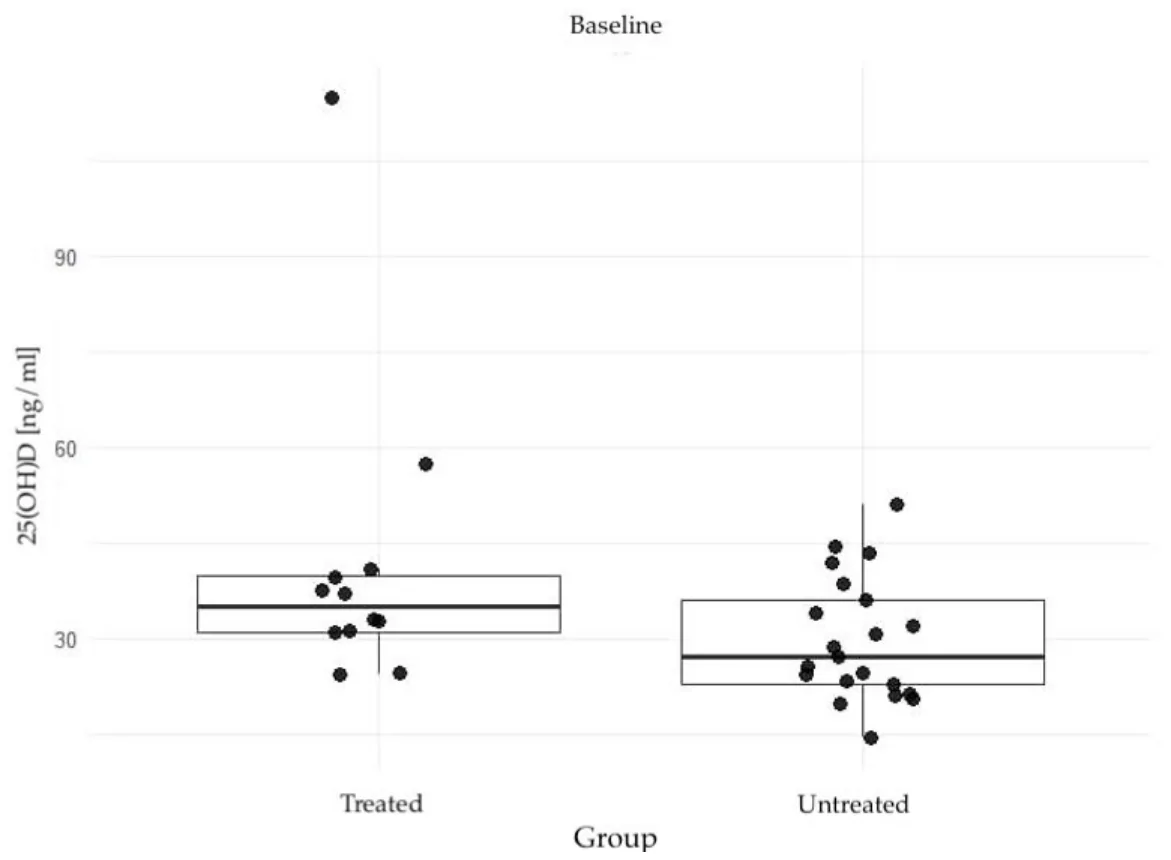
Comparison of 25(OH)D concentrations based on targeted therapy status.

**Figure 4:**
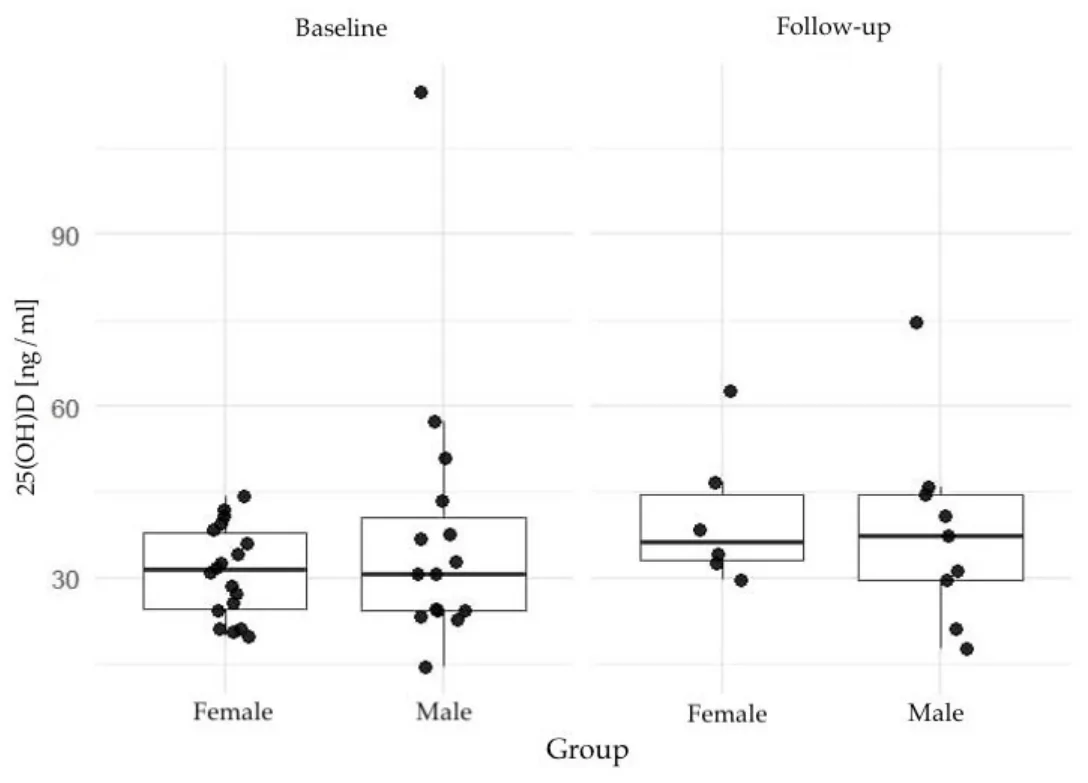
Comparison of 25(OH)D concentrations in female and male participants at baseline and after 12 months of follow-up.

**Figure 5:**
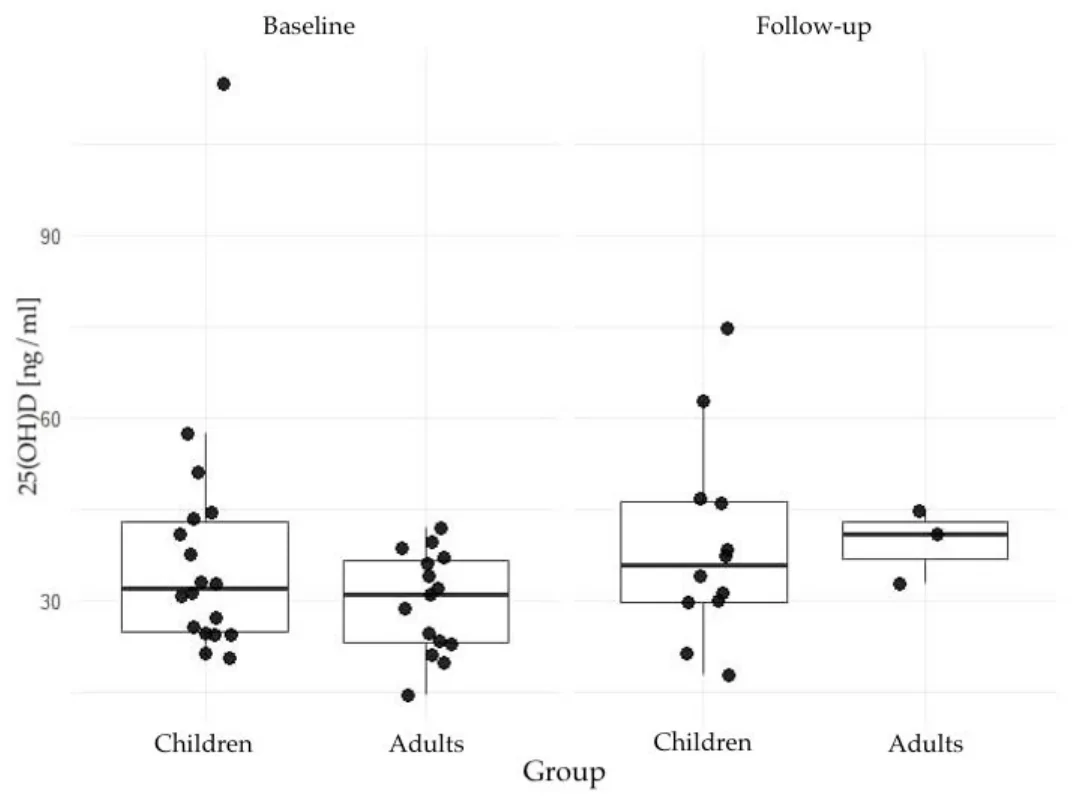
Comparison of 25(OH)D concentrations by age group at baseline and after 12 months of follow-up.

**Figure 6:**
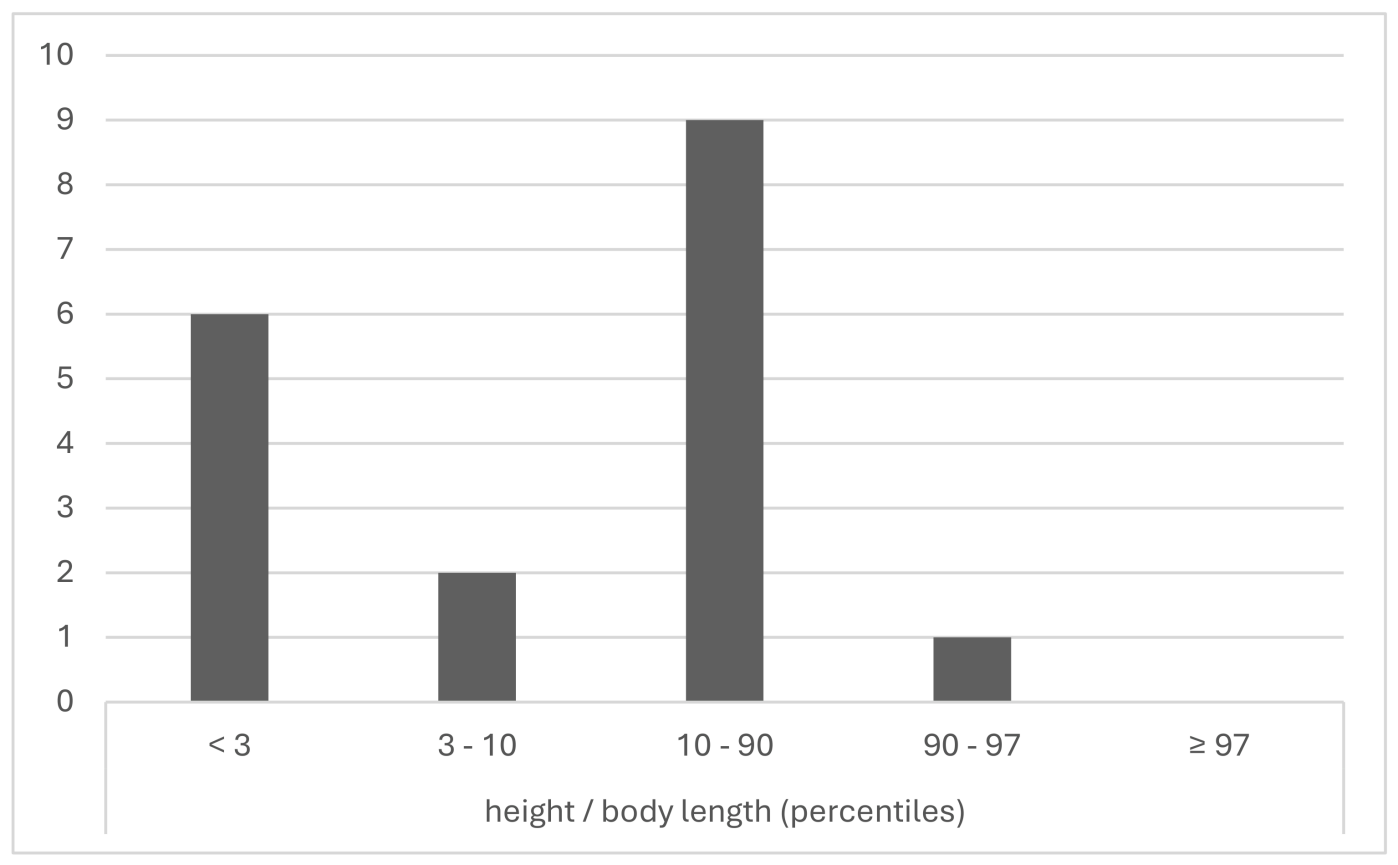
Distribution of height and body length in a pediatric population with hypophosphatasia by percentile range (n = 18).

**Figure 7:**
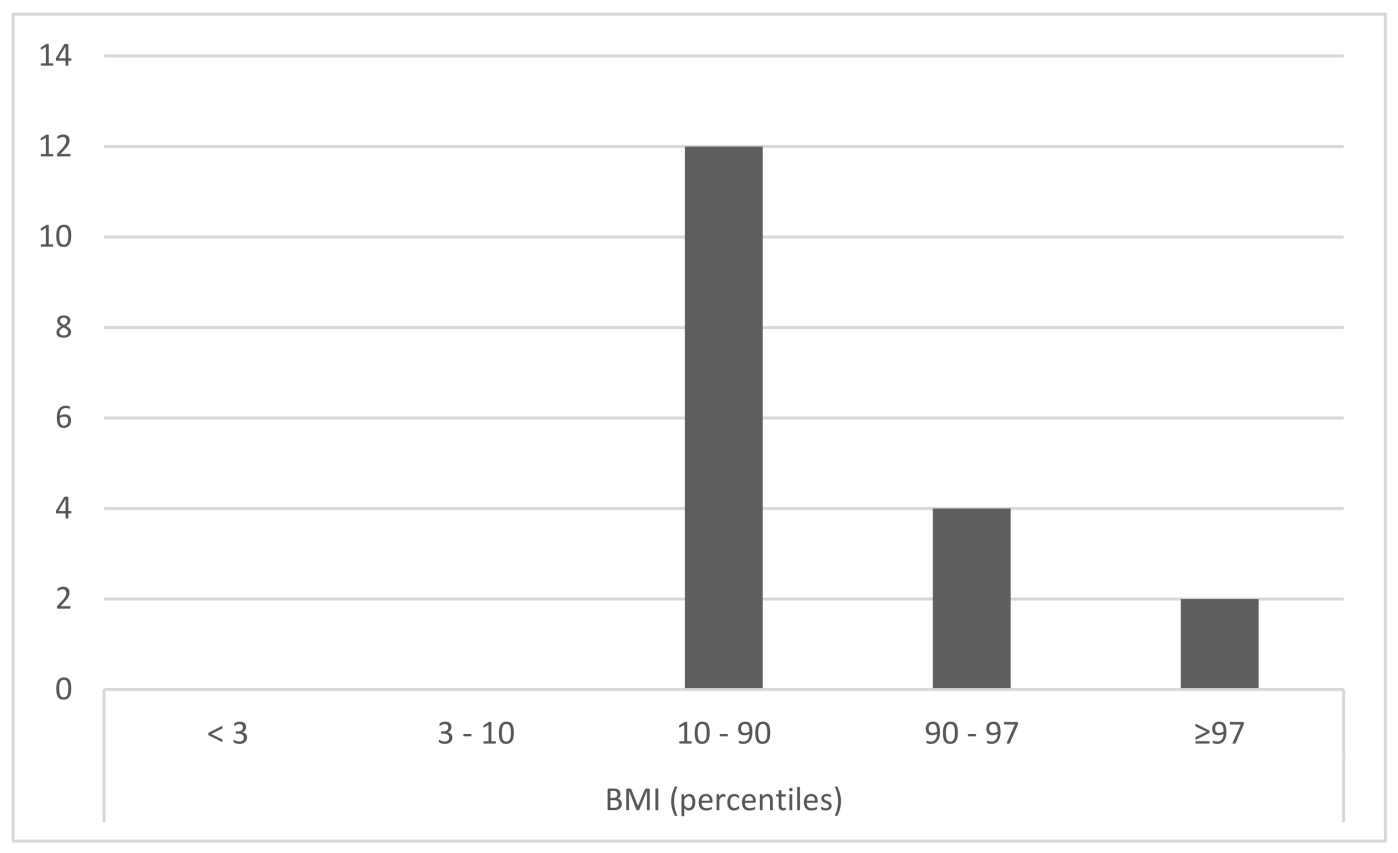
Distribution of BMI in a pediatric population with hypophosphatasia by percentile range (n = 18).

**Table 1:** Changes in vitamin D levels in patients receiving ERT and vitamin D supplementation.

ID	Vit. D (Baseline)	Vit. D (Follow-Up)	Difference Between Vit. D Concentrations	
HPP_01		46.8		
HPP_02		34.1		
HPP_03		29.7		
HPP_04	40.8	38.4	2.4	↓
HPP_05	24.6	17.6	7	↓
HPP_06	24.4	21.2	3.2	↓
HPP_07	32.9	31.2	1.7	↓
HPP_08	31.1	62.8	31	↑
HPP_09	36.9	44.6	7.7	↑
HPP_10	37.6	37.3	0.3	↓
HPP_11	30.8	40.9	10.1	↑
HPP_12	32.7	29.8	2.9	↓
HPP_13	114.9	45.9	69	↓
HPP_14	39.6	32.7	6.9	↓
HPP_15	57.4	74.6	17.2	↑
HPP_16	21.2			

The numerical values represent the absolute difference in vitamin D levels between baseline and follow-up, while the arrows (↑, ↓) indicate whether the concentration increased or decreased.

**Table 2:** Vitamin D concentrations in all patients at baseline and follow-up, by age group.

Patient ID	Age Group	Vit. D Baseline (ng/mL)	Vit. D Follow-Up (ng/mL)
HPP_01	Child	-	46.8
HPP_02	Child	-	34.1
HPP_03	Child	-	29.7
HPP_04	Child	40.8	38.4
HPP_05	Child	24.6	17.6
HPP_06	Child	24.4	21.2
HPP_07	Child	32.9	31.2
HPP_08	Child	31.1	62.8
HPP_09	Adult	36.9	44.6
HPP_10	Child	37.6	37.3
HPP_11	Adult	30.8	40.9
HPP_12	Child	32.7	29.8
HPP_13	Child	114.9	45.9
HPP_14	Adult	39.6	32.7
HPP_15	Child	57.4	74.6
HPP_16	Child	21.2	-
HPP_17	Adult	36	-
HPP_18	Child	30.7	-
HPP_19	Adult	34.1	-
HPP_20	Adult	14.4	-
HPP_21	Child	50.9	-
HPP_22	Child	44.3	-
HPP_23	Adult	21.1	-
HPP_24	Adult	22.9	-
HPP_25	Adult	24.5	-
HPP_26	Adult	38.6	-
HPP_27	Adult	23.4	-
HPP_28	Adult	31.9	-
HPP_29	Child	20.6	-
HPP_30	Child	43.5	-
HPP_31	Adult	28.7	-
HPP_32	Child	25.6	-
HPP_33	Child	24.4	-
HPP_34	Adult	19.8	-
HPP_35	Child	27.2	-
HPP_36	Adult	41.9	-

**Table 3:** Changes in serum calcium, phosphorus, and magnesium concentrations between baseline and follow-up.

Test Parameter (Unit)	Starting Point (Baseline) Mean (Min–Max)	After 12 Months (Follow-Up) Mean (Min–Max)	*p*-Value
**Calcium** (mmol/L)	2.9 (1.90–2.66)	2.55 (2.47–2.67)	0.104
**Phosphorus** (mmol/L)	1.58 (0.94–2.22)	1.74 (1.09–2.20)	0.515
**Magnesium** (mmol/L)	0.85 (0.76–0.98)	0.86 (0.77–0.98)	0.753

**Table 4:** Assessment of changes in serum vitamin D concentrations after 12 months of follow-up in the matched group of patients (n = 12).

Test Parameter (Unit)	Starting Point (Baseline) Median (Q1–Q3)	After 12 Months (Follow-Up) Median (Q1–Q3)	Δ Value (Follow-Up -Baseline) Median (Q1–Q3)	*p*-Value (Wilcoxon Test)
Vit. D (ng/mL)	34.90 (31.03–39.90)	37.85 (30.85–44.93)	−2.05 (−4.13–8.30)	0.969

**Table 5:** Spearman’s rank correlation analysis of vitamin D concentrations and selected clinical, biochemical, and anthropometric parameters at baseline and follow-up, as well as an assessment of the dynamics of changes (Δ).

Clinical Variable Tested	Sample Size (n)	Spearman’s r	*p*-Value (Nominal)	*p*-Value (FDR *)
Baseline—correlation with 25(OH)D concentration at baseline				
Urinary Ca/Cr ratio	13	−0.692	0.009	0.087
Serum inorganic phosphate	33	0.376	0.031	0.156
BMI	15	0.336	0.221	0.879
Total serum calcium	32	0.181	0.320	0.754
Urinary calcium	13	−0.275	0.364	0.754
Serum magnesium	31	0.156	0.401	0.754
BMI (percentile)	15	0.221	0.428	0.879
Patient age	33	−0.119	0.509	0.754
Alkaline phosphatase (ALP)	33	0.107	0.552	0.754
Height/body length (percentile)	15	−0.161	0.566	0.879
Urinary creatinine	13	0.159	0.603	0.754
Parathyroid hormone (PTH)	32	−0.068	0.711	0.791
Body weight	15	0.102	0.718	0.879
After 12 months (follow-up)—correlation with 25(OH)D concentration at follow-up				
Body weight	12	0.622	0.031	0.184
Serum inorganic phosphate	15	−0.486	0.066	0.281
Height/body length	12	0.524	0.080	0.240
Patient age	15	0.448	0.094	0.281
Height/body length (percentile)	12	−0.423	0.170	0.262
BMI	12	0.420	0.175	0.262
Alkaline phosphatase (ALP)	15	−0.204	0.467	0.934
BMI (percentile)	12	0.189	0.557	0.668
Serum magnesium	15	−0.048	0.864	0.995
Parathyroid hormone (PTH)	15	0.032	0.909	0.995
Total serum calcium	15	−0.002	0.995	0.995
Rate of change—correlation with the change in vitamin D concentration (Δ)				
Change in magnesium concentration (Δ Mg)	12	−0.271	0.394	0.829
Change in inorganic phosphate concentration (Δ P)	12	−0.203	0.527	0.829
Change in parathyroid hormone concentration (Δ PTH)	12	0.137	0.672	0.829
Change in calcium concentration (Δ Ca)	12	0.127	0.695	0.829
Change in alkaline phosphatase activity (Δ ALP)	12	−0.070	0.829	0.829

* FDR (False Discovery Rate), the Benjamini–Hochberg correction method for multiple testing.

## Data Availability

The original contributions presented in this study are included in the article.
